# A Long-Lost Coffee Bean Tastes as Good as the Best—To Understand Why, Scientists Turn to Chemistry

**DOI:** 10.1021/acscentsci.5c01454

**Published:** 2025-08-18

**Authors:** Marta Zaraska

## Abstract

The climate-resistant bean boasts a chemical profile similar to Arabica’s.

## Graphical abstract

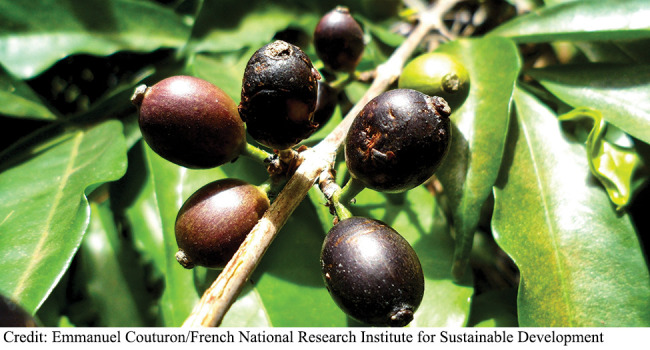

When Aaron Davis, a botanist at the Royal Botanic Gardens, Kew, first tried stenophylla coffee, his expectations were low. But the first sip took him by surprise: “I was like, oh my goodness, that is just unbelievable,” he says. The coffee was sweet, with notes of chocolate and caramel and a hint of jasmine.

Stenophylla had been lost to consumers since the early 20th century and only recently rediscovered by Davis and his colleagues in the rain forests of Sierra Leone. To Davis and a panel of judges, it tasted like Rwandan Bourbon—a superior variety of Arabica. From a chemical perspective, that similarity was puzzling.

**Figure:**
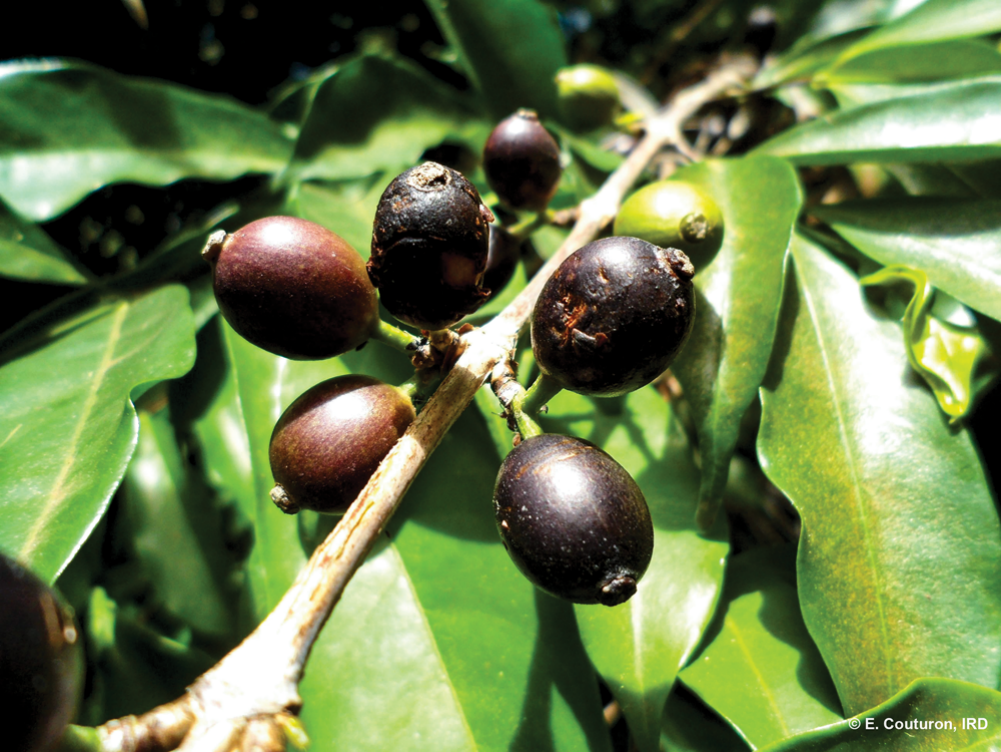
Black fruits of stenophylla coffee. Credit: Emmanuel Couturon/French National Research Institute for Sustainable Development.

*Coffea stenophylla* is a separate species from both *Coffea arabica* and *Coffea canephora (robusta).* “It occurs in a completely different environment. It’s got black fruits instead of red fruits,” Davis says. “If you’re a coffee nut, it’s kind of mind blowing.”

To find out why it tastes so much like Arabica, Davis and his colleagues set out to research stenophylla’s chemistry. The results were published in March: despite being a different species, stenophylla has a similar molecular profile to Arabica’s. Both have comparable levels of bitter and even fruity compounds that make Arabica taste great. But, as Davis and his colleagues found out, stenophylla also has some unique qualities—for example, in addition to caffeine, it contains the rare stimulant theacrine.

The study’s results are “really exciting,” says Elizabeth Tomasino, an enologist and coffee researcher at Oregon State University, who was not involved in the study. The findings, she says, are a step toward understanding the chemistry behind how different species give the qualities we like in a brew—and identifying stenophylla as a potential alternative to Arabica could help coffee production adjust to climate change.

With increasing droughts and rising temperatures putting pressure on coffee production (by 2050, the global area suitable for coffee may shrink by half), growers and researchers are on the lookout for more climate-change-resistant species and varieties. Robusta, the second most common species after Arabica, is quite, well, robust—but it just doesn’t taste as good. Stenophylla, on the other hand, can flourish at much higher temperatures than Arabica, and among over 130 known species of coffee, it’s the only one that matches Arabica in quality.

**Figure:**
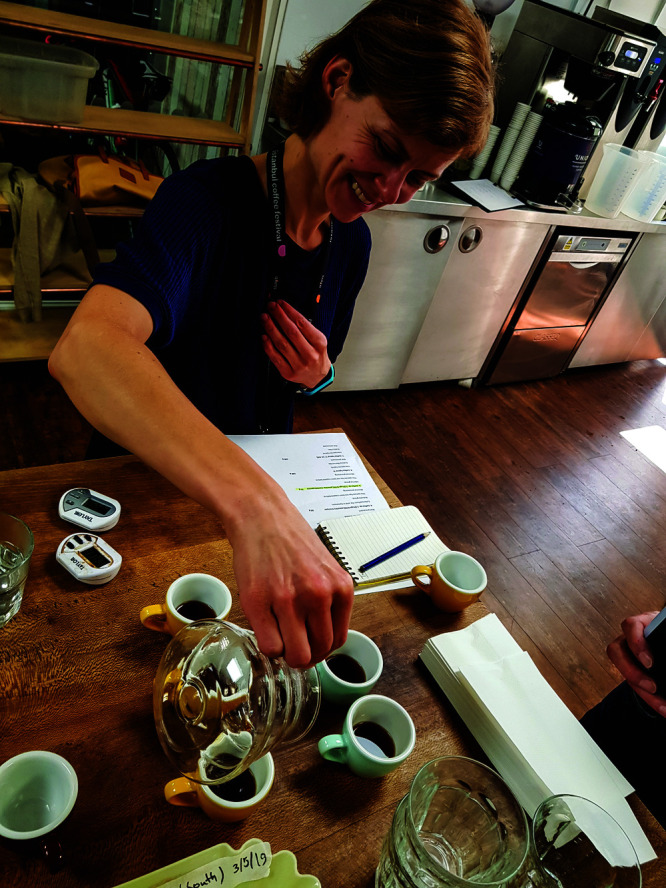
Edita Chodarcevic, a certified coffee judge and head of quality at Union Hand-Roasted Coffee, prepares samples for tasting and scoring brewed coffee for quality in September 2020. Credit: Union Hand-Roasted Coffee.

Many factors matter for how coffee will taste and smell when we drink it—such as where the plant grew, how much it rained, and how the beans were stored. But roasting is a big one: “If you ever smelled a raw coffee bean, it would not have the same character at all as roasted coffee does,” says Devin Peterson, a food scientist at the Ohio State University.

During roasting, the sucrose in the bean caramelizes, which is how coffee gets its brown color. Some amino acids and peptides degrade in the Strecker process, while other amino acids and sucrose go through the Maillard reaction, darkening the bean even further as delicious smells waft.

Over 1,000 volatile organic compounds have been identified in roasted coffee. 2-Methylpyrazine smells nutty; 2,3-hexanedione smells creamy. And as the temperature rises, the chemistry changes, meaning that brewing method matters too. In an espresso, for example, the concentration of 3-hexanone goes up with temperature, giving a sweet, fruity aroma. Even the shape and texture of the cup can influence how testers perceive the brew.

To figure out how stenophylla produces such an excellent flavor, and one so similar to Arabica’s, Davis and his colleagues opted to compare the chemical makeup of the beans. But this is not so simple a feat.

“Coffee is a hugely complex matrix,” Tomasino says. This is why the official coffee-tasting lexicon includes such words as *pineapple*, *tobacco*, *petroleum*, *jasmine*, and even *skunky*. There are over 700 compounds in unroasted beans that make up the flavor and aroma of coffee. Many of the compounds, Tomasino says, aren’t important for how we perceive our beverage; others, meanwhile, are disproportionately so. “Compounds at super teeny tiny concentrations can be hugely impactful,” she says.

**Figure:**
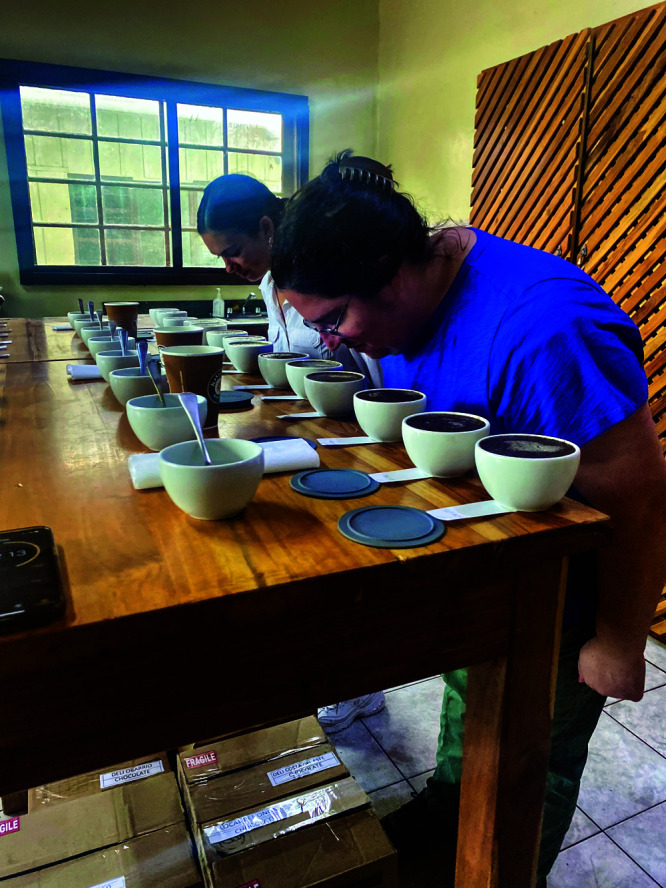
Oregon State University’s Elizabeth Tomasino (right) and her student Victoria Koyner partake in a coffee cupping exercise at Kotowa Farms in Panama in December 2024. Credit: Diana Batista Ledezma.

To learn more about the chemical compositions of coffee beans, Davis and his colleagues focused on the unroasted kind—“the roasting process introduces so much variation,” says Eliot Jan-Smith, a chemist at the Royal Botanic Gardens, Kew, and one of the study’s authors. The team finely crushed unroasted beans of stenophylla, Arabica, and robusta species; macerated the powders in a solution of methanol and water; and then put them through liquid chromatography. The researchers found many similarities between Arabica and stenophylla, such as in their content of citric acid. “Acidity in coffee is a really big predictor of quality,” Jan-Smith says.

Stenophylla and Arabica also had similar levels of chlorogenic acids, while robusta had more, which could explain its lower flavor ratings. “Some of these compounds might be linked to astringency: that’s the feeling of drying out on the inside of the cheeks,” Jan-Smith says.

Another similarity between stenophylla and Arabica was in their amounts of trigonelline—one of the most abundant alkaloids in coffee and an important contributor to bitterness—and sucrose. “Generally, a sweeter cup of coffee, or more naturally sweet, is considered a good thing,” Jan-Smith says.

But the two beans did not completely share a chemical profile. The most striking difference was the presence of theacrine in stenophylla—a first in a coffee bean. A cousin to both caffeine and theobromine (found in chocolate), theacrine’s main source so far has been kucha, a tea endemic to China. The compound is touted by some as a great mood-enhancer and a physical performance booster, yet research so far is mixed. “There are some who say it gives you energy, keeps you awake, there are some who say it helps you sleep,” Jan-Smith says.

For Jan-Smith, the theacrine in stenophylla may have other benefits: namely, it could serve as a marker to detect fake batches. With only a few plots of the coffee growing so far, mostly in Sierra Leone, stenophylla “is going to be expensive initially, and so it’s going to be an obvious target for fraud,” he says.

**Figure:**
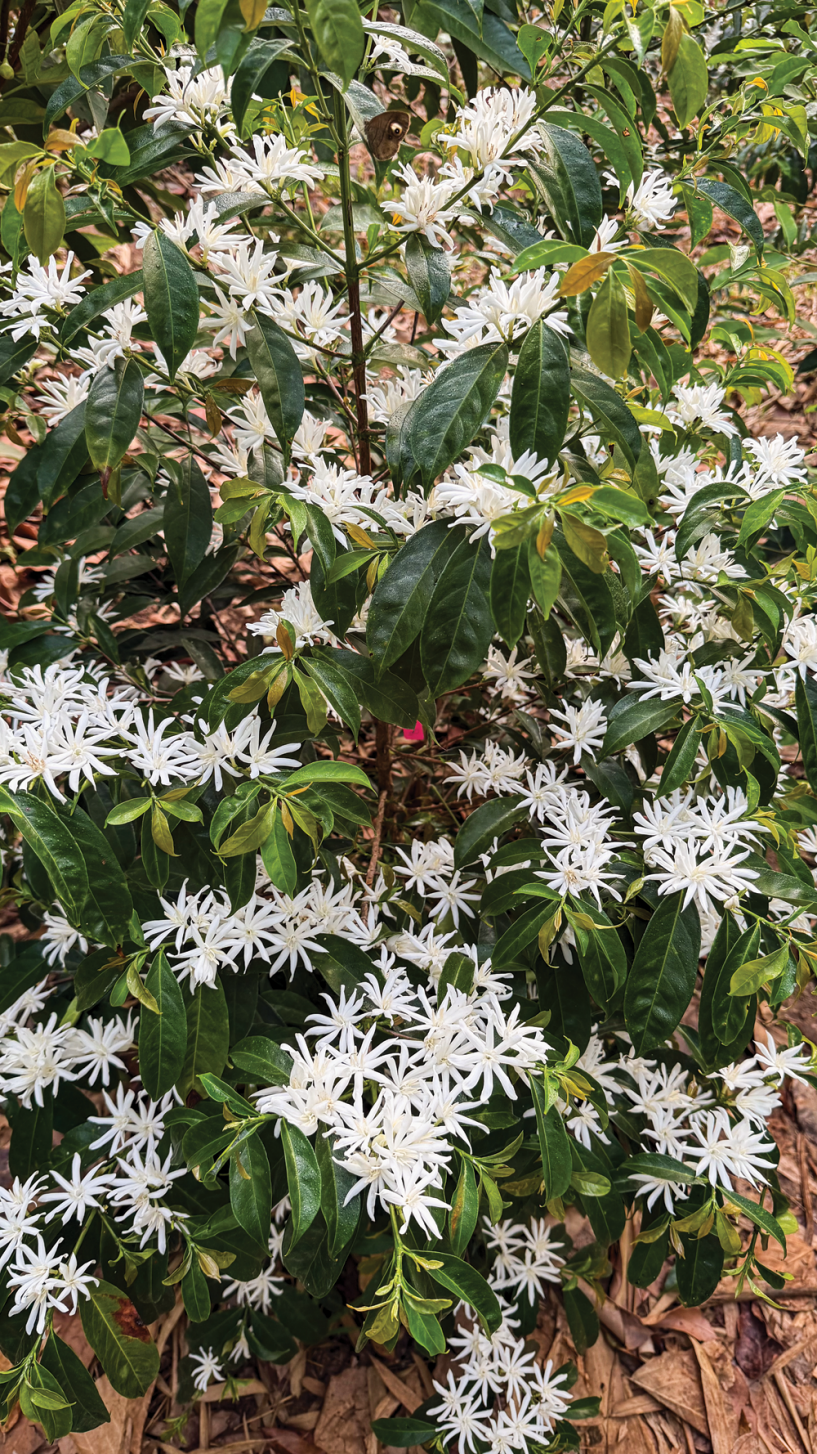
A stenophylla coffee plant in bloom. Credit: South India Coffee Company.

Other studies confirm that certain molecules can be used as chemical fingerprints to prevent coffee adulteration—for example, to distinguish varieties of Arabica such as Bourbon or Typica, which are considered superior. For stenophylla, that marker could be theacrine. Checking samples for such markers “might well be more rapid and more feasible than genetics,” Jan-Smith says.

Finding what makes coffee taste great, Davis says, is vital for climate-proofing the crop. “Most of the breeding work has been for yield, disease resistance, pest resistance. Now we have to breed coffees that still have all those things—and still taste great,” he says. And decoding coffee’s chemistry is the vital first step.

*Marta Zaraska is a freelance contributor to* Chemical & Engineering News, *the independent news publication of the American Chemical Society.*

